# Extramarital Sex Prevalence, Associated Factors, and Viral Load Among People Living With HIV/AIDS Attending the Antiretroviral Therapy (ART) Clinic in Central Hospital, Sapele, Delta State, Nigeria: A Cross-Sectional Study

**DOI:** 10.7759/cureus.115842

**Published:** 2026-09-05

**Authors:** Chinagozi C Ahaiwe, Caleb C Anulaobi, Sylvester I Okafor, Fejiro V Obodo, Esther Udensi, Hezekiah Ihejirika, Hilda Afakwu-Adimoha

**Affiliations:** 1 School of Public Health, University of Port Harcourt, Port Harcourt, NGA; 2 Department of Medicine and Surgery, Abia State University, Uturu, NGA; 3 Department of Internal Medicine, Central Hospital, Sapele, NGA; 4 Department of Medicine, Abia State University, Uturu, NGA; 5 Department of Surgery, NES Healthcare, Buckinghamshire, GBR; 6 Department of Community Medicine, Federal Medical Center, Yenagoa, NGA

**Keywords:** extramarital sex, nigeria, plhiv, prevalence, viral load

## Abstract

Background

Risky sexual behaviors such as extramarital sex are associated with an increased risk of HIV transmission and poor treatment outcomes. Common factors such as religion, masculinity, societal norms, women's economic hardship, income, alcohol, marital conflict, and sexual dissatisfaction are associated with extramarital sex, leading to increased transmission of HIV. This study aimed to determine the prevalence of extramarital sex, associated factors, clinical stages of disease, and viral load outcomes among people living with HIV (PLHIV)

Methods

This was a descriptive cross-sectional study conducted among PLHIV attending the antiretroviral therapy (ART) clinic at Central Hospital, Sapele, Delta State, Nigeria. The chi-square bivariate test was used to assess the association between the dependent and independent variables, with a p-value <0.05 considered statistically significant. Odds ratios with 95% confidence intervals (CIs) were reported to indicate the strength of the associations.

Results

The study included a sample of 216 participants, with a mean age of 48.0 ± 12.4 years and females being 73.1% (158). The prevalence of extramarital sexual activities among PLHIV was 10.2% (22), with common reasons such as curiosity and excitement, opportunity or temptation, emotional dissatisfaction, sexual dissatisfaction, seeking revenge, and lack of intimacy stated. Almost all the participants had either an undetectable viral load result (178, 82.4%) or a result <100 copies/ml (31, 14.4%). The male respondents were found to be six times more likely to engage in extramarital sex than females (OR = 5.97; 95% CI: 2.35-15.14; p-value <0.001). Those who considered extramarital sex to be acceptable were 25 times more likely to engage in it (OR = 24.67; 95% CI: 8.43-72.15; p < 0.001).

Conclusion

This study provided insight into the prevalence of extramarital sex among PLHIV, their clinical characteristics, and viral load patterns. It showed that factors associated with extramarital sex include cultural, societal, and structural factors. Efforts channeled towards comprehensive sexuality education and behavioral modification will be effective in reducing risky sexual behaviors.

## Introduction

Human immunodeficiency virus (HIV) continues to be a disease of public health concern despite the availability of antiretroviral therapy (ART), prompt diagnosis, and prevention strategies [1]. Approximately 40.8 million people are living with HIV worldwide, with sub-Saharan Africa having a major burden of the disease [2] and a prevalence of 1.4% among adults in Nigeria [3]. However, HIV-related morbidity and mortality have drastically reduced due to the widespread scale-up of ART, making it a manageable chronic condition and resulting in a longer life expectancy and improved quality of life for people living with HIV (PLHIV), with treatment outcomes routinely monitored through routine viral load assays [4]. A viral load result of <1000 copies/ml, referred to as viral load suppression, is associated with reduced disease progression, reduced morbidity and mortality, and the elimination of transmission risk [5]. Several factors result in poor adherence to medications, resulting in unsuppressed viral loads and increased transmission risk, with one of these factors being risky sexual behaviors such as extramarital sex. Viral outcomes are influenced by behavioral, psychosocial, and treatment factors. Extramarital sex often coexists with behavioral and psychosocial factors that affect adherence to treatment and continuation in care. Concerns about disclosure, perceived stigma, and relationship stress, amongst others, can potentially hinder medication adherence, preventing viral suppression [6,7].

Extramarital sex is described as engaging in sex with someone other than one's spouse or stable partner. It can also be described as cheating, sexual concurrency, or an extramarital affair. It is known to increase the risk of HIV infection progressively. New infections often stem from unprotected heterosexual intercourse, including extramarital sex encounters [8,9]. Common factors include religion, masculinity, societal norms, women's economic hardship, income, alcohol, marital conflict, and sexual dissatisfaction. Individuals having sexual relationships outside of their marriages may contribute to the transmission of HIV, especially when there is no use of barrier contraceptive measures or the application of other preventive strategies. Extramarital sexual relationships are shown to occur more frequently among men than women [10].

In Nigeria, although significant progress has been made in expanding access to ART and preventive services, there are still challenges with treatment adherence, retention in care, and prevention, with efforts being channeled towards preventive services such as condom use, pre-exposure and post-exposure prophylaxis, disclosure practices, and behavioral modifications, which are essential in reducing the transmission of HIV. Social and cultural factors influence sexual behaviors, and they vary across geographical settings and healthcare facilities. For instance, social and cultural norms restrict women from interfering in the sexual lives and practices of their husbands. They are made to believe that they are expected to remain monogamous, while the men are at liberty to engage in polygamy or extramarital sexual relationships [11]. These circumstances encourage risky behaviors and poor adherence to ART, potentially worsening the control of HIV transmission and treatment outcomes.

Considering the potential effects of extramarital sexual activity on HIV transmission and treatment outcomes, there is a need to understand the occurrence and determinants among PLHIV on ART. This study aimed to determine the prevalence of extramarital sex, the associated factors, clinical characteristics, and viral load among PLHIV attending an ART clinic in a central hospital in Delta State, Nigeria.

## Materials and methods

Study design and setting

We conducted a descriptive cross-sectional study at the antiretroviral therapy (ART) clinic of the Central Hospital, Sapele, Delta State. The clinic caters to approximately 3,300 active persons living with HIV. The study participants were recruited from November 2025 to February 2026. Delta State is a state in Nigeria created on August 27, 1991, with Asaba as the capital [12]. University of Port Harcourt Research Ethics Committee approved the study (approval number: UPH/SPH/ACAD/MSC/ETHICS/2025/220).

Study population

The study population included persons living with HIV attending and receiving HIV/AIDS-related care at the ART clinic of the Central Hospital, Sapele, Delta State. It included male and female sexually active clients who gave their consent and were aged between 18 and 65 years. Those who were too ill to participate or unable to respond to questions were excluded from the study.

Sample size determination and sampling techniques

Using Cochran’s formula [13], with an estimated proportion of 16.2% in Nigeria [14], a margin of error of 5%, and a confidence interval of 95%, a minimum sample size of 209 participants was determined. A multistage sampling technique was used to select the study participants. The first stage involved purposive sampling to select the two major clinic days (Tuesdays and Thursdays) when clients mainly attend the ART clinic. The second stage involved stratified sampling of the clients into old and new clients. The third stage involved simple random sampling to select eligible persons for recruitment into the study.

Data collection and analysis

Primary data were collected using questionnaires and proformas (see Appendices), while secondary data were obtained from study participants’ clinic folders. Trained research assistants collected data. The questionnaire was self-administered to study participants in a private setting to ensure privacy and confidentiality. No identifying details were collected, and participants were encouraged to complete the questionnaires independently. Upon completion of data collection, the data were checked for completeness and accuracy. The questionnaire was pretested using 10% of the participants, ensuring completeness, clarity, acceptability, and suitability. Data entry and cleaning were performed in Microsoft Excel (Microsoft Corporation, Redmond, USA), and the data were then exported to IBM SPSS version 23 (IBM Corp., Armonk, USA) for statistical analysis. The dependent variable was the practice of extramarital activity among respondents, while the independent variables were the sociodemographic characteristics of the respondents as well as their viral load and clinical staging status.

Descriptive statistics, including the analyzed data, were expressed as frequencies and percentages for categorical data and mean + SD and mode for numerical data and were presented in tables and charts. The chi-square bivariate test was used to assess the association between the dependent and independent variables and identify the presence of a relationship. A simple unadjusted odds ratio from 2x2 tables was then calculated to identify the magnitude of the association between the variables. A p-value of <0.05 was considered statistically significant, and the odds ratios with 95% confidence intervals were reported to indicate the strength of associations.

## Results

Socio-demographic characteristics of the respondents

Of the 230 questionnaires distributed, 216 were retrieved and fully completed, yielding a response rate of 94%. The socio-demographic characteristics of the respondents are presented in Table 1. The majority of the respondents were aged 40 to 49 years (71, 32.9%), with a mean age of 48.0 ± 12.4 years. Most were female (158, 73.1%), self-employed (141, 65.3%), and Christians (208, 96.3%). Most had attained a secondary level of education as their highest level of education (113, 52.3%), earned between 2000 and 3000 Nigerian naira daily (117, 54.2%), and were married (113, 52.3%).

**Table 1:** Socio-demographic characteristics of the respondents (n = 216) NGN: Nigerian naira

Variable	Category	Frequency, n (%)
Age (years)	18 – 29	10 (4.6)
	30 – 39	45 (20.8)
	40 – 49	71 (32.9)
	50 – 59	51 (23.6)
	≥60	39 (18.1)
Sex	Male	58 (26.9)
	Female	158 (73.1)
Occupation	Self-employed	141 (65.3)
	Employed by others	54 (25.0)
	Not employed	21 (9.7)
Religion	Christianity	208 (96.3)
	Islam	6 (2.8)
	Traditional	2 (0.9)
Level of education	None	9 (4.2)
	Primary	34 (15.7)
	Secondary	113 (52.3)
	Tertiary	52 (24.1)
	Postgraduate	8 (3.7)
Daily income (NGN)	<500	12 (5.6)
	500 – 1000	24 (11.1)
	1000 – 2000	57 (26.4)
	2000 – 3000	117 (54.2)
	>3000	3 (1.4)
Marital status	Single	35 (16.2)
	Cohabiting	5 (2.3)
	Married	113 (52.3)
	Separated	19 (8.8)
	Divorced	3 (1.4)
	Widowed	41 (19.0)

Sexual history and characteristics of the respondents

The results of the sexual history and characteristics of the respondents showed that most of them had been involved in sexual intercourse in the past (175, 81.0%). The majority first engaged in sexual intercourse between the ages of 20 and 29 years (85, 48.6%), with a mean age of 18.9 ± 8.0 years. 36 (20.6%) of those who had engaged in sexual intercourse in the past had two or more sexual partners, while 17 (9.7%) had more than one sexual partner at the time of responding. Almost all of those who were sexually active engaged in vaginal sex (175, 99.4%), and almost half had never used any means of protection during sex (74, 42.3%). Only 88 (40.7%) of the respondents had ever been tested for sexually transmitted infections (STIs), with the majority being tested more than a year ago (65, 73.9%) (Table 2).

**Table 2:** Characteristics, perception, practices, viral outcomes and clinical staging of respondents (n = 216)

Variable	Category	Frequency, n (%)
Ever had sexual intercourse	Yes	175 (81.0)
	No	41 (19.0)
Age of sexual debut, years (n = 175)	10 – 19	59 (33.7)
	20 – 29	85 (48.6)
	30 – 39	12 (6.9)
	Cannot remember	19 (10.9)
Number of past sexual partners (n = 175)	≤2	139 (79.4)
	>2	36 (20.6)
Number of present sexual partners (n = 175)	≤1	158 (90.3)
	>1	17 (9.7)
Type of sexual activity engaged in (multiple responses)	Vaginal	175 (99.4)
	Anal	1 (0.6)
	Oral	0 (0.0)
Use of protection during sex (n = 175)	Always	32 (18.3)
	Sometimes	47 (26.9)
	Rarely	22 (12.6)
	Never	74 (42.3)
Ever been tested for STIs	Yes	88 (40.7)
	No	87 (40.3)
	Do not know	41 (19.0)
If yes, when was the last screening (n = 88)	Past 6 months	13 (14.8)
	6 months – 1 year ago	10 (11.4)
	More than a year ago	65 (73.9)
Ever been diagnosed with STIs	Yes	14 (6.5)
	No	202 (93.5)
STIs experienced (multiple answers) (n = 14)	Chlamydia	1 (7.1)
	Gonorrhea	7 (50.0)
	Herpes	1 (7.1)
	Syphilis	4 (28.6)
	Others (yeast)	1 (7.1)
Consider extramarital sex to be acceptable	Yes	8 (3.7)
	No	195 (90.3)
	Sometimes, depending on the situation	13 (6.0)
Reasons why extramarital sex should be acceptable (n = 21)	No emotional fulfillment in marriage	1 (4.8)
	No physical/sexual satisfaction in marriage	4 (19.0)
	Seeking variety/new partners	3 (14.3)
	Seeking validation/attention	1 (4.8)
	Marital problems/conflicts	12 (57.1)
Extramarital sex can be justified if both parties in a marriage are not meeting each other’s needs	Yes	16 (7.4)
	No	175 (81.0)
	Sometimes, depending on the situation	25 (11.6)
Engaged in extramarital sex	Yes	22 (10.2)
	No	194 (89.8)
Frequency of engagement (n = 22)	1 time	5 (22.7)
	2 – 5 times	8 (36.4)
	6 – 10 times	6 (27.3)
	>10 times	3 (13.6)
Reasons for engaging in extramarital sex (multiple reasons)	Emotional dissatisfaction in marriage	3 (8.6)
	Sexual dissatisfaction in marriage	6 (17.1)
	Opportunity/temptation	9 (25.7)
	Seeking revenge or payback	1 (2.9)
	Curiosity/excitement	11 (31.4)
	Lack of intimacy in marriage	4 (11.4)
	Other reasons	1 (2.9)
Last viral load (copies/ml)	Undetectable (<20)	148 (68.5)
	20 – 99	47 (21.8)
	100 – 1000	4 (1.9)
	>1000	17 (7.9)
Current viral load (copies/ml)	Undetectable (<20)	178 (82.4)
	20 – 99	31 (14.4)
	100 – 1000	4 (1.9)
	>1000	3 (1.4)
Clinical staging	I	194 (89.8)
	II	18 (8.3)
	III	4 (1.9)
	IV	0 (0.0)
Opportunistic infections	Present	8 (3.7)
	Absent	208 (96.3)
Types of opportunistic infections (n = 8)	Tuberculosis	8 (100.0)
	Others	0 (0.0)

Perception of extramarital sexual practices among the respondents

Eight (3.7%) respondents considered extramarital sex to be acceptable, while 13 (6.0%) considered it sometimes, depending on the situation. Marital problems and conflicts (12, 57.1%) were considered to be the major reason for these considerations. Sixteen (7.4%) of them considered that extramarital sex can be justified if both parties in a marriage are not meeting each other’s needs, while 25 (11.6%) considered it to be sometimes justifiable, depending on the situation (Table 2).

Prevalence of extramarital sex activities among respondents

The prevalence of extramarital sexual activities among the study respondents was 22 (10.2%). The largest proportion of these respondents had engaged in extramarital activities between two and five times (8, 36.4%), with their common reasons being curiosity and excitement (11, 31.4%) and the opportunity or temptation (9, 25.7%) to engage in extramarital activities (Table 2).

Disease characteristics of the respondents

In terms of the characteristics of the disease of the respondents, the majority (148, 68.5%), as at their last viral load sample assay a year ago, had an undetectable viral load result, followed by 47 (21.8%) having a result of less than 100 copies/ml. Then, on assessment of their current viral load, almost all the respondents had either an undetectable viral load result (178, 82.4%) or a result less than 100 copies/mL (31, 14.4%). The clinical staging assessment showed that most of the respondents were in clinical stage 1 (194, 89.8%), with only eight (3.7%) having opportunistic infections, which were only tuberculosis (Table 2).

Factors associated with extramarital sex practice among the respondents

In this study, Pearson’s chi-square test assessed the factors associated with involvement in extramarital sexual activities. The factors included the sex of the respondents, their present number of sexual partners, and their acceptance, as well as the justification of engagement in extramarital sexual activities based on unmet marital needs (Table 3).

**Table 3:** Factors associated with extramarital sex practice among respondents Pearson’s chi-square (χ²) was used to assess the factors associated with involvement in extramarital sexual activities. Significant at P-value < 0.05 r: referent; OR: Odds ratio; 95% CI: Confidence interval; NGN: Nigerian naira; STI: sexually-transmitted infection

Variable	Engaged in extramarital sex			Test statistic	P-value	OR	95% CI
	Yes, n (%)	No, n (%)	Total, n (%)	X^2^			
Age (years)							
≤48	10 (8.6)	106 (91.4)	116 (100.0)	0.670	0.413	0.69^r^	0.29 – 1.68
>48	12 (12.0)	88 (88.0)	100 (100.0)				
Total	22 (10.2)	194 (89.8)	216 (100.0)				
Sex							
Male	14 (24.1)	44 (75.9)	58 (100.0)	16.874	<0.001	5.97^r^	2.35 – 15.14
Female	8 (5.1)	150 (94.9)	158 (100.0)				
Total	22 (10.2)	194 (89.8)	216 (100.0)				
Marital status							
Married	15 (13.3)	98 (86.7)	113 (100.0)	2.472	0.116	2.10^r^	0.82 – 5.38
Unmarried	7 (6.8)	96 (93.2)	103 (100.0)				
Total	22 (10.2)	194 (89.8)	216 (100.0)				
Education							
≤Secondary	16 (10.3)	140 (89.7)	156 (100.0)	0.003	0.955	1.03^r^	0.38 – 2.77
>Secondary	6 (10.0)	54 (90.0)	60 (100.0)				
Total	22 (10.2)	194 (89.8)	216 (100.0)				
Daily income (NGN)							
>2000	11 (9.2)	109 (90.8)	120 (100.0)	0.306	0.580	0.78^r^	0.32 – 1.89
≤2000	11 (11.5)	85 (88.5)	96 (100.0)				
Total	22 (10.2)	194 (89.8)	216 (100.0)				
Occupation							
Unemployed	3 (14.3)	18 (85.7)	21 (100.0)	0.428	0.513	1.54^r^	0.42 – 5.73
Employed	19 (9.7)	176 (90.3)	195 (100.0)				
Total	22 (10.2)	194 (89.8)	216 (100.0)				
Number of sexual partners							
≤1	11 (7.0)	147 (93.0)	158 (100.0)	19.471	<0.001	0.11^r^	0.03 – 0.34
>1	7 (41.2)	10 (58.8)	17 (100.0)				
Total	22 (10.2)	194 (89.8)	216 (100.0)				
Previous STI diagnosis							
Yes	16 (9.4)	155 (90.6)	171 (100.0)	0.616	0.433	0.67^r^	0.25 – 1.83
No	6 (13.3)	39 (86.7)	45 (100.0)				
Total	22 (10.2)	194 (89.8)	216 (100.0)				
Current viral load							
Undetectable	17 (9.6)	161 (90.4)	178 (100.0)	0.445	0.505	0.70^r^	0.24 – 2.02
Detectable	5 (13.2)	33 (86.8)	38 (100.0)				
Total	22 (10.2)	194 (89.8)	216 (100.0)				
Extramarital sex can be acceptable							
Yes	12 (57.1)	9 (42.9)	21 (100.0)	56.071	<0.001	24.67^r^	8.43 – 72.15
No	10 (5.1)	185 (94.9)	195 (100.0)				
Total	22 (10.2)	194 (89.8)	216 (100.0)				
Extramarital sex can be justified if marital needs are not being met							
Yes	17 (41.5)	24 (58.5)	41 (100.0)	54.121	<0.001	24.08^r^	8.14 – 71.26
No	5 (2.9)	170 (97.1)	175 (100.0)				
Total	22 (10.2)	194 (89.8)	216 (100.0)				

Male respondents were found to be six times more likely to engage in extramarital sexual activities than their female counterparts (OR = 5.97; 95% CI: 2.35-15.14; p-value <0.001). Respondents who considered having extramarital sexual relations acceptable were 25 times more likely to engage in extramarital sexual activities than those who do not consider it to be acceptable (OR = 24.67; 95% CI: 8.43-72.15; p < 0.001). Lastly, those who responded that extramarital sex can be justified if marital needs are not being met were 24 times more likely to engage in extramarital sexual activities than those who responded otherwise (OR = 24.08; 95% CI: 8.14-71.26; p < 0.001).

## Discussion

This study aimed to determine the prevalence and associations of extramarital sex among PLHIV attending the ART clinic of Central Hospital, Sapele, as well as their clinical staging status and viral load. The findings show valuable insights into the characteristics of extramarital sex among PLHIV. The study showed that about one in ten persons had engaged in extramarital sexual activities, mostly due to curiosity and excitement. Most of the respondents also had undetectable viral load counts. The factors associated with involvement in extramarital sexual activities were the sex of the respondents, the present number of sexual partners, and the justification of engaging in extramarital sex on the grounds of unmet marital needs.

The sexual history of the respondents showed the mean age of first sexual encounter to be 18.9 ± 8.0 years, and almost half (85, 48.6%) of the respondents reported having their first sex between ages 20 and 29 years. Close to half of those who were sexually active had never used any form of protection during sex. This predisposes their partners to STIs, including HIV. The finding of poor condom use is similar to a study conducted in southwestern Nigeria among people visiting a friendly HIV counseling and testing center, which shows 45.2% (n=74) of participants not using a condom in their last sexual encounter [15].

The prevalence of extramarital sex among the respondents living with HIV was 10.2%, with most engaging in sexual activities out of curiosity or the excitement they experienced during the activity. This is an important finding indicating behaviors that increase the risk of spreading STIs. This is consistent with other studies that show the persistent habit of extramarital sexual activity despite the existence of cultural and religious deterrents to involvement in such activities. These studies, however, found different levels of prevalence of extramarital sex and other risky sexual behaviors [7,16,17]. Most of the respondents were sexually active, with 9.7% having more than one sexual partner and not using any condoms or using them inconsistently, showing the considerable risk of STIs that the population is exposed to. Respondents in our study reported reasons for engaging in extramarital sex to include unmet marital needs, emotional dissatisfaction, marital conflicts, curiosity, and excitement. These reasons were also reported in similar studies where people reported various factors that predispose them to engaging in extramarital activities or risky behaviors [9,10]. In circumstances of this nature, married individuals or those with partners become convergence points who transfer STIs, including HIV, causing the risk of resistant HIV or HIV epidemics. This shows the need for comprehensive education for PLHIV to be aware of the potential implications of engaging in extramarital sexual activities. This education can also be extended to the general population to discourage the persistent involvement of individuals in risky sexual behaviors.

Concerning the clinical staging of the disease state of the respondents, this study showed that most were classified as being at clinical stage I of disease progression, and a small proportion experienced tuberculosis as an opportunistic infection. This suggests asymptomatic states or early disease phases with good medication adherence or good response to treatment. It can also portray the benefits of early diagnosis and access to care, which ensures that PLHIV are enrolled in care early before immune suppression kicks in. In HIV management, clinical staging is an important tool for monitoring disease severity and assessing prognosis and treatment decisions, especially in regions with limited access to laboratory facilities. If these individuals in clinical stage I do not continuously adhere to their medications, they are likely to progress to advanced clinical stages of the disease [18].

The study showed most respondents had undetectable viral load results, and a few respondents had suppressed or unsuppressed results. An undetectable viral load has been shown to have no risk of transmission of HIV. However, individuals with low-level viremia have a likelihood of persistent replication of the virus, leading to a risk of drug resistance, which may eventually defeat the effectiveness of treatment [19]. Individuals with low-level viremia or an unsuppressed viral load are capable of transmitting the virus to their sexual partners, especially when no form of protective barrier such as a condom is used during sexual activity [15]. This set of PLHIV represents a subset who may be encountering interruptions in treatment, poor adherence, or emerging drug resistance, and who, with no timely intervention, can progress to advanced stages of the disease. This necessitates optimized treatment, adherence counseling, and routine monitoring of viral load among PLHIV on ART [19].

The factors associated with extramarital sex were the sex of the respondents, their acceptance of engagement in extramarital sex, and the justification that it was based on unmet marital needs. The male respondents were six times more likely to engage in extramarital sex than the female respondents. Those who considered extramarital sex acceptable were 25 times more likely to engage in extramarital activities. Also, those who felt that having extramarital activities can be justified if marital needs are not being met were 24 times more likely to engage in the activities. These findings show the multidimensional nature of extramarital sexual activity and its associations. The gender preponderance in extramarital sexual activity is consistent with previous studies, which show the level of activity to be significantly higher in males than females [7,9,10,16]. This is influenced by factors such as social and cultural norms, where there is more permissiveness about men engaging in these activities, and women's involvement is stigmatized. The increased rate of extramarital sexual activity among those who consider it acceptable gives credence to the role of partner concurrency as a determinant of the persistent prevalence of HIV [10]. The justification of engaging in extramarital sex on the grounds of unmet marital needs increases the likelihood of engaging in the activity. This creates a situation that makes the behavior acceptable to the individual. Addressing these behaviors through comprehensive sexuality education and health promotion will be effective in reducing the prevalence of HIV and other STIs.

Limitations

This study used a cross-sectional study design and so has limitations in establishing causal associations. The study population was hospital-based and self-reported and therefore is prone to selection and recall bias. Limitations also include the number of study participants, which was just a little above the minimum sample size, the lack of adjustments for potential confounders in the analysis, and potential bias, as this was a study of social sensitivity. Further research can utilize a prospective study to ascertain the association between extramarital sexual activities and viral load outcomes and other treatment characteristics of PLHIV.

## Conclusions

This study assessed the prevalence of extramarital sexual activity among PLHIV, providing insight into their clinical characteristics and viral load outcomes. It explained the sexual behaviors of PLHIV and the various associations of extramarital sex in the context of a state in southern Nigeria. It provides explanations for the individual characteristics, as well as structural, cultural, and social factors that sustain risky sexual behaviors and their interplay with clinical outcomes of PLHIV. There is a need for government agencies to prioritize comprehensive approaches that integrate behavioral interventions into the routine management of PLHIV and the propagation of public health campaigns and awareness to address risky sexual behaviors.

## Appendices

Questionnaire

Extramarital Sex Prevalence, Associated Factors, and Viral Load among People Living with HIV/AIDS Attending the ART Clinic in Central Hospital, Sapele, Delta State, Nigeria.

You are invited to participate in a study on extramarital sex prevalence, associated factors, and viral load among people living with HIV/AIDS attending the ART clinic in Central Hospital, Sapele, Delta State, Nigeria.

Confidentiality:

All information collected during this study will be kept confidential. Your responses to the questionnaire will be anonymous.

Please complete all sections to the best of your ability.

CONSENT:

I hereby consent to participate in this research. I understand that my participation is voluntary, and I am free to withdraw at any time without any consequences.

o   Yes

o   No

SECTION 1: Socio-demographic characteristics

1.     What is your age as of your last birthday?

……………………………….

2.     What is your sex?

o   Male

o   Female

3.     What is your occupation?

o   Self-employed

o   Employed by others

o   Not employed

4.     What is your religion?

o   Christianity

o   Islam

o   Traditional

5.     What is your level of education?

o   No formal education

o   Primary

o   Secondary

o   Tertiary

o   Postgraduate

6.     What is your daily income in Nigerian naira?

o   Less than 500

o   500 - 1000

o   1000 - 2000

o   2000 - 3000

o   More than 3000

7.     What is your marital status?

SECTION 2: Sexual history and characteristics of respondents

8.     Have you ever had sexual intercourse?

o   Yes

o   No

If No, skip to Question 16

9.     What was the age you had your first sex?

…………………………………………..

10.  What is the number of your past sexual partners?

…………………………………………...

11.  What is the number of your present sexual partners?

……………………………………………

12.  What type of sexual activity do you engage in? (Multiple responses)

      Vaginal sex

      Anal sex

      Oral sex

13.  How often do you use protection during sex?

o   Always

o   Sometimes

o   Rarely

o   Never

14.  Have you ever been tested for sexually transmitted infections?

o   Yes

o   No

o   Do not know

15.  If yes, when was the last testing done?

o   Past 6 months

o   6 months - 1 year ago

o   More than a year ago

16.  Have you ever been diagnosed with sexually transmitted infections?

o   Yes

o   No

17.  If yes, what sexually transmitted infections have you had? (multiple responses)

      Chlamydia

      Gonorrhea

      Herpes

      Syphilis

      Others …… (specify)

SECTION 3: Perception of extramarital sex relationships

18.  Do you consider extramarital sexual relationships to be acceptable?

o   Yes

o   No

19.  If yes, what are the reasons why extramarital sex should be acceptable? (multiple responses)

      Sometimes, depending on the situation

      No emotional fulfilment in marriage

      No physical/sexual satisfaction in marriage

      Seeking variety or new partners

      Seeking validation or attention

      Marital problems or conflicts

20.  Do you think that extramarital sex can be justified if both parties in a marriage is not meeting each other’s needs?

o   Yes

o   No

o   Sometimes, depending on the situation

SECTION 4: Practices of extramarital sexual relationships

21.  Have you ever engaged in extramarital sex?

o   Yes

o   No

22.  If yes, how many times?

o   Once

o   2 - 5 times

o   6 - 10 times

o   More than 10 times

23.  What was your reason for engaging in extramarital sex? (multiple responses)

      Emotional dissatisfaction in marriage

      Sexual dissatisfaction in marriage

      Opportunity or temptation

      Seeking revenge or payback

      Curiosity or excitement

      Lack of intimacy in marriage

      Others …….. (specify)

Data extraction form

Participant identification

1.     Patient ID: ……………….

2.     Date of data extraction: …………….

Viral load data

3.     Last documented viral load result (copies/ml): …………………………..

4.     Current documented viral load result (copies/ml): …………………………….

Clinical staging of disease

5.     WHO Clinical staging:

o   Stage I

o   Stage II

o   Stage III

o   Stage IV

6.     Opportunistic infection:

o   Present

o   Absent

7.     If yes, type of opportunistic infection: …………………….
